# Mechanistic Insights into *Vernonia calvoana*-Induced Apoptosis in Ovarian Cancer Cells via the Intrinsic Pathway

**DOI:** 10.3390/ijms27177887

**Published:** 2026-09-03

**Authors:** Ariane M. Chitoh, Clement G. Yedjou, Ingrid K. Tchakoua, Sylvianne Njiki, Felicite K. Noubissi, Titilope Komolafe, Kayode Komolafe, Oluwatoyin V. Odubanjo, Paul B. Tchounwou

**Affiliations:** 1 Research Center for Health Disparities Research and Innovation (RCMI), Jackson State University, 1400 Lynch Street, Jackson, MS 39217, USA; felicite.noubissi_kamdem@jsums.edu (F.K.N.); titilope.r.komolafe@jsums.edu (T.K.); kayode.c.komolafe@jsums.edu (K.K.); oluwatoyin.v.odubanjo@jsums.edu (O.V.O.); 2 Department of Biology, College of Science, Engineering and Technology, Jackson State University, 1400 Lynch Street, Jackson, MS 39217, USA; ingrid.k.tchakoua@students.jsums.edu; 3 Department of Biological Sciences, Florida Agricultural and Mechanical University, Tallahassee, FL 32307, USA; clement.yedjou@famu.edu (C.G.Y.); sylvianne.njiki@famu.edu (S.N.); 4 Research Center for Urban Health Disparities Research and Innovation (RCMI), Morgan State University, 1700 E. Cold Spring Lane, Baltimore, MD 21251, USA

**Keywords:** *Vernonia calvoana*, OVCAR-3, apoptosis, mitochondrial membrane potential, DNA laddering, caspase-3, caspase-9, Bcl-2, p53, cytochrome c, ovarian cancer, natural product, health disparity

## Abstract

*Vernonia calvoana* (VC), a commonly used medicinal plant in West Africa, has been shown by our research team to inhibit the proliferation of OVCAR-3 ovarian cancer cells through mechanisms involving oxidative stress, DNA damage, and S-phase cell cycle arrest. The objective of the current study was to elucidate the intrinsic apoptotic mechanisms triggered by VC fraction seven (VCF7). OVCAR-3 cells were treated with VCF7 (0, 8, 16, and 32 μg/mL) for a duration of 48 h. Apoptosis was assessed using Annexin V/Propidium Iodide (PI) staining followed by flow cytometry analysis. Mitochondrial membrane potential (ΔΨm) was assessed through JC-1 staining and confocal microscopy, while chromatin condensation was analyzed using DAPI staining. DNA fragmentation was examined by agarose gel electrophoresis. Caspase 3 activity was measured using flow cytometry. Protein expression levels of p53, Bcl-2, cytochrome c, caspase-9, and caspase-3 were determined by Western blot analysis, and mRNA expression levels of p53 and Bcl-2 were evaluated using qRT-PCR. VCF7 induced apoptosis in a concentration-dependent manner. Analysis using Annexin V/PI indicated an increase in apoptotic cell populations from 10.5% to 30%, along with a rise in necrotic cells from 7% to 50% across treatment concentrations. A modest, concentration-associated decrease in mitochondrial membrane potential was recorded (0.96-, 0.88-, and 0.85-fold at 8, 16, and 32 μg/mL, respectively; *p* < 0.05). DAPI staining validated the concentration-dependent chromatin condensation and nuclear fragmentation. The analysis of DNA fragmentation showed progressive internucleosomal degradation, appearing as a smear pattern with distinct fragments at elevated concentrations, indicative of concurrent apoptotic and necrotic cell death. The activation of caspase-3 reached a peak of 28% at 16 μg/mL. Western blot analysis indicated an upregulation of p53, a downregulation of Bcl-2, an increase in total cytochrome c protein levels, and an increased expression of caspase-9 and caspase-3 in a concentration-dependent manner. These findings were corroborated at the transcriptional level by qRT-PCR, which showed increased p53 mRNA and decreased Bcl-2 mRNA expression. Taken together, these results underscore the potential of VCF7 as a promising plant-derived anticancer agent and support the need for further preclinical and clinical studies in ovarian cancer.

## 1. Introduction

Ovarian cancer (OC) is the most lethal gynecological malignancy worldwide and represents a major global health burden. According to the most recent GLOBOCAN 2022 data, approximately 324,603 women were newly diagnosed with OC globally, with 206,956 deaths reported in that year alone [1]. The disease ranks fourth among female cancer causes of death worldwide and accounts for 4.3% of global female cancer deaths, surpassing all other gynecological cancers [2]. Projections from the World Ovarian Cancer Coalition and the International Agency for Research on Cancer (IARC) indicate that annual incidence will approach 500,000 cases and mortality will exceed 350,000 deaths by 2050, with disproportionate increases projected in Africa and Asia [3,4]. Despite advances in surgical and systemic therapy, the overall 5-year survival rate for OC remains approximately 48–49% in high-income settings and substantially lower in low- and middle-income countries, largely because over 70% of cases are diagnosed at an advanced stage [5]. OC also represents a striking model of racial health disparity. For African American (AA) women, the 5-year relative survival rate for OC in the United States is reported at 41%, as opposed to 49% for Non-Hispanic White (NHW) women [6,7]. Moreover, the racial gap has persisted despite advances in standard-of-care treatment strategies [7]. A meta-analysis of 16 studies involving 190,107 subjects identified an 18% higher mortality risk among Black women as compared to White women with a relative risk (RR) of 1.18 (95% confidence interval [CI] 1.11–1.26) [8]. Such racial inequality can be detected even in healthcare systems where all people have equal access to medical services, as in the case of the U.S. Military Health System [8,9]. Factors responsible for such outcomes include disparities in access to specialists in gynecologic oncology, dose reductions in the administration of chemotherapeutics, low engagement in clinical trials, structural racism, and possibly differences in tumor biological behavior [10,11].

In the initial treatment approach for OC, maximal surgical debulking is combined with platinum–taxane (carboplatin/paclitaxel) chemotherapy and a PARP inhibitor in BRCA mutations [12]. However, despite encouraging initial success, around 70% of patients will experience recurrence within two years because of acquired platinum resistance [13]. Moreover, current chemotherapeutic agents are characterized by severe toxicity profiles, including alopecia, neurotoxicity, nephrotoxicity, myelosuppression, and fertility impairment, thus necessitating novel, effective, and safe options [14,15,16,17]. Plant extracts have been actively investigated for the treatment of cancer due to their unique composition and multitargeting potential, low safety concerns, and cheap production [18,19].

*Vernonia calvoana*, also known as sweet bitter leaves, has been shown to possess antioxidative, anti-inflammatory, hepatoprotective, hypoglycemic, and hypolipidemic properties [20,21,22,23,24]. Our previous research demonstrated that VCF7 causes a significant growth inhibition of the human ovarian cancer OVCAR-3 cell line (IC_50_ = 18.56 μg/mL), and induces oxidative stress via lipid peroxidation, along with catalase and glutathione peroxidase depletion, DNA double-strand breakage, and cell cycle arrest [25]. The results suggest that VCF7 may possess potential antineoplastic activity. The goal of this current study was to examine the pathways by which VCF7 induces apoptosis. Apoptosis, or programmed cell death, is mainly regulated by two pathways: extrinsic (death-receptor) and intrinsic (mitochondrion-dependent) pathways [26]. The mitochondrial pathway, which is induced by intracellular stress signals regulated by Bcl-2 family proteins, whose ratio determines the decision of survival versus death [27]. The depolarization of the mitochondrial membrane potential (Ψm) causes the first release of cytochrome c into the cytoplasm. Secondly, the activation of caspase-9 is followed by caspase-3. Finally, nuclear chromatin condensation and fragmentation with internucleosomal DNA cleavage, representing the hallmarks of apoptosis, are detectable via a “ladder” on agarose gel electrophoresis [28,29,30,31]. The p53 protein is a tumor suppressor and a key transcription factor upstream of the apoptosis cascades, responding to DNA and oxidative stress by activating pro-apoptotic genes of the Bcl-2 family while inhibiting anti-apoptotic genes [32,33,34]. The present study aimed to evaluate the intrinsic mitochondrial pathway by which VCF7 induces apoptosis in ovarian cancer cells. In other words, the objective of this research is to characterize the sequence of key molecular events leading to the apoptosis of OVCAR-3 cells exposed to VCF7. To the best of our knowledge, this present study is the first to investigate the intrinsic mitochondrial apoptotic pathway induced by *Vernonia calvoana* in ovarian cancer cells. It moves beyond the antiproliferative and DNA-damaging effects observed in our previous report to define the specific downstream apoptotic cascade from mitochondrial membrane depolarization, cytochrome c release, caspase 9/3 activation, and p53/Bcl2 protein expression through which VCF7 induces cell death. Furthermore, it also advances a locally sourced medicinal plant as a potential candidate for anticancer activity and supports the development of accessible, low-cost, plant-derived therapies as alternative options for underserved populations.

## 2. Results

### 2.1. VCF7 Induces Dose-Dependent Apoptosis and Necrosis in OVCAR-3 Cells (Annexin V/PI)

**Figure 1:**
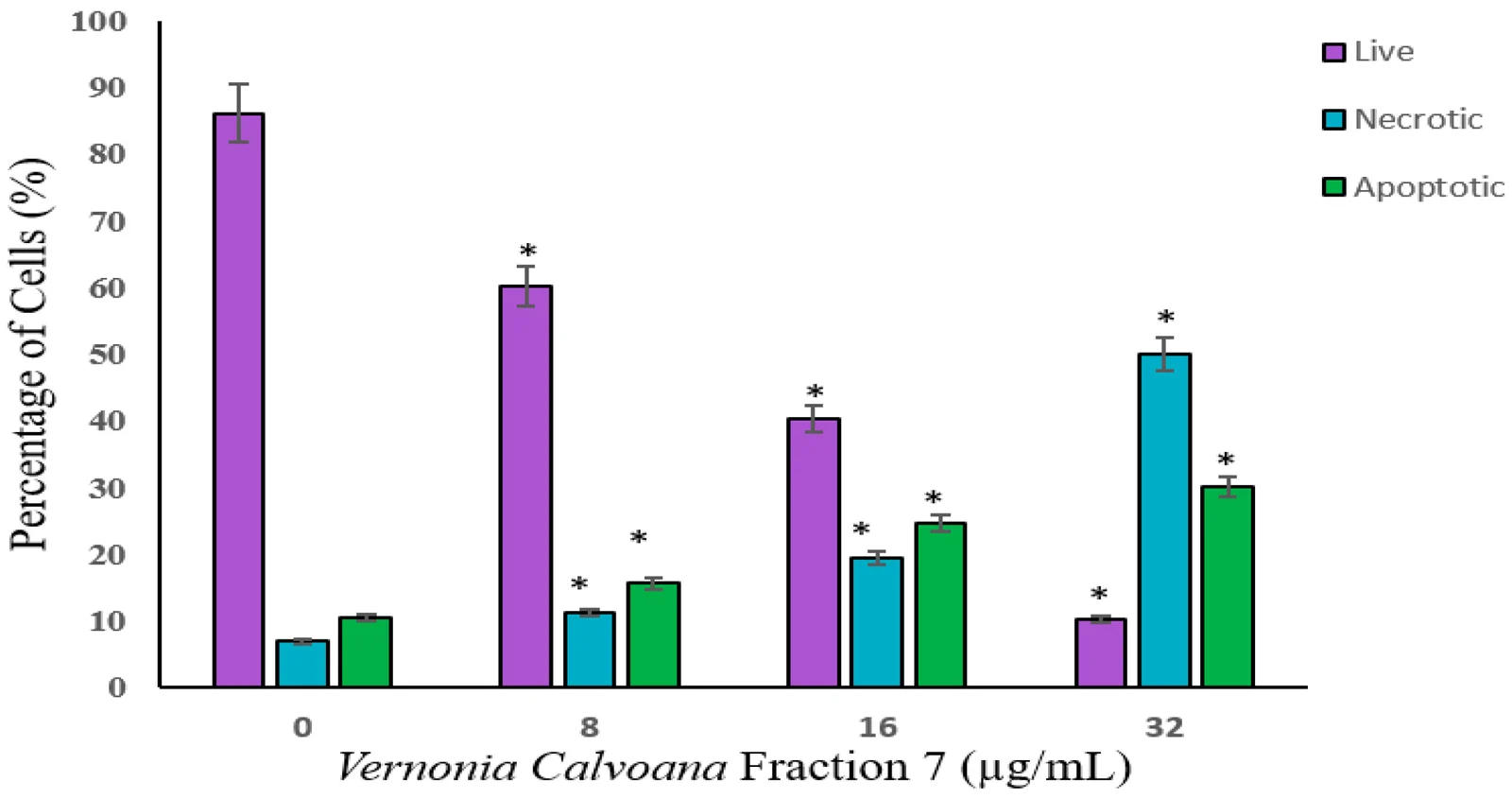
Percentages of live, necrotic, and apoptotic cells. Data points represent the average percentages of 3 independent experiments. * Denotes a statistically significant difference (*p* < 0.05) between the control and the treated groups.

To quantitatively distinguish apoptotic from necrotic cell death, Annexin V-FITC/PI double-staining flow cytometry was performed on OVCAR-3 cells treated with VCF7 (0, 8, 16, 32 μg/mL) for 48 h. Annexin V detects early phosphatidylserine externalization on the outer plasma membrane of the cell, which signals an early apoptotic phase, while PI staining identifies cells with disrupted membrane integrity (late-stage apoptotic or necrotic). As shown in Figure 1, VCF7 treatment produced a clear, statistically significant dose-concentration-dependent induction of cell death. The percentages of apoptotic (Annexin V^+^/PI^−^) cells were 10.5%, 15.63%, 25%, and 30% at 0, 8, 16, and 32 μg/mL VCF7, respectively (Figure 1). Concomitantly, necrotic (PI^+^) cell populations increased from 7% (control) to 11%, 20%, and 50% at 8, 16, and 32 μg/mL, respectively (Figure 1). The viable cell fraction declined correspondingly from ~82.5% in untreated controls to ~20% at 32 μg/mL. Statistical analysis showed that all treated groups were statistically significantly different from the control (*p* < 0.05; one-way ANOVA with Dunnett’s post hoc test).

### 2.2. VCF7 Causes Mitochondrial Membrane Potential Depolarization in OVCAR-3 Cells

**Figure 2:**
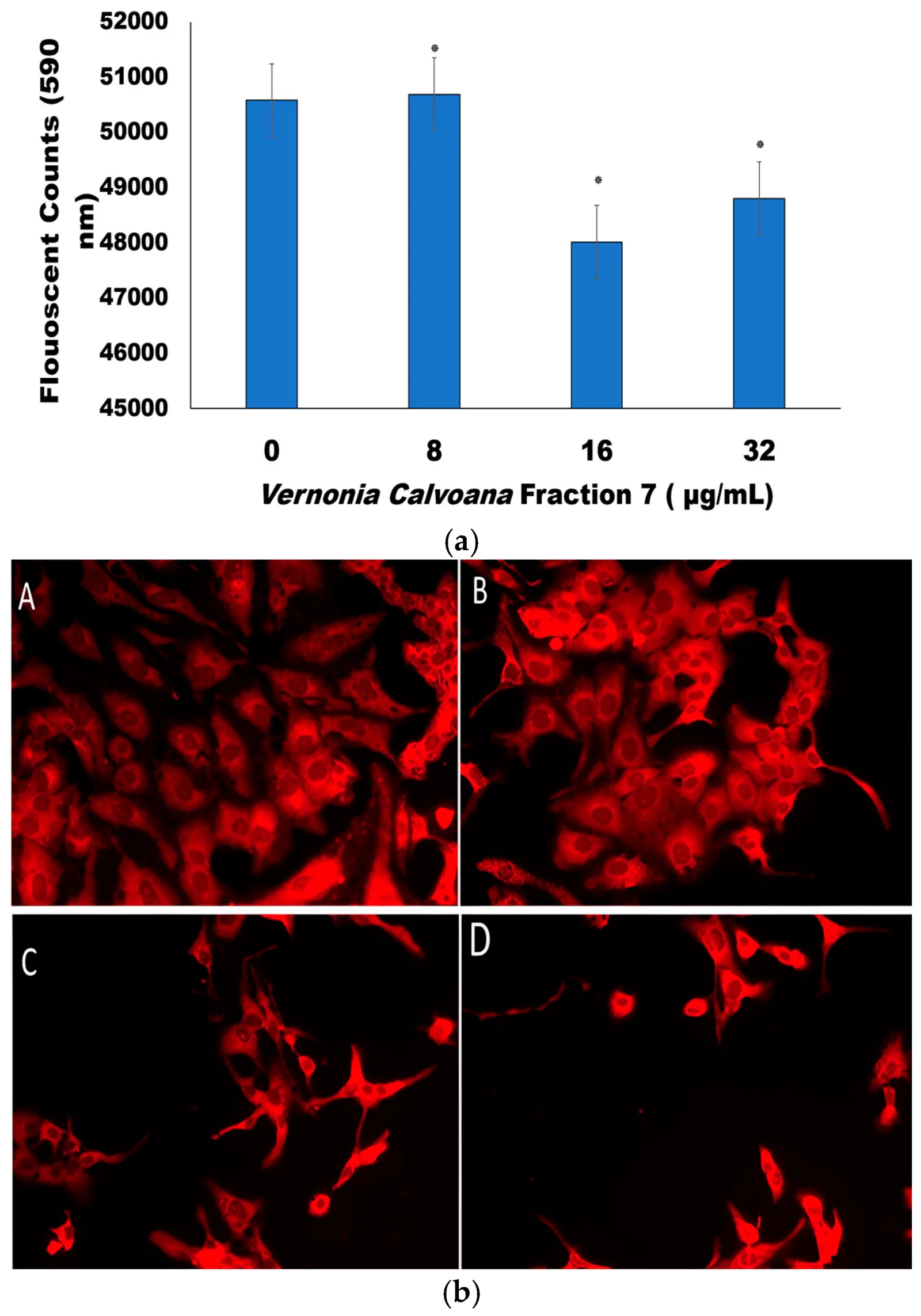
(**a**) The effect of VCF7 on mitochondrial membrane depletion in OVCAR-3 cells. Data represent the means ± SDS of three independent experiments (*n* = 3). * Indicates a statistically significant (*p* < 0.05) difference when comparing the control and treated samples using one-way ANOVA. (**b**) Representative fluorescence images of JC1-stained OVCAR-3 cells, untreated or treated with VC F7. (**A**) Control, (**B**) 8 µg/mL, (**C**) 16 µg/mL, (**D**) 32 µg/mL. All fluorescence images were captured under 20× optical resolution of the microscope.

Mitochondrial membrane potential (Ψm) is one of the early markers of apoptosis induction. Disruption of Ψm precedes and initiates the release of pro-apoptotic factors, including cytochrome c from the mitochondrial intermembrane space [33]. We assessed Ψm using the ratio metric JC-1 dye, which forms red-fluorescent J-aggregates in mitochondria with intact high Ψm (Figure 2b) and shifts to green-fluorescent monomers upon Ψm depolarization. Figure 2a shows the fluorescence intensity of JC-1 dye at 590 nm. The intensity was significantly reduced relative to the control when treated with VCF7, showing a concentration-dependent decrease (Figure 2a). The decreases in J-aggregate fluorescence counts were 0.96-, 0.88-, and 0.85-fold for 8 μg/mL, 16 μg/mL, and 32 μg/mL, respectively, compared to the control (0 μg/mL), as shown in Figure 2a. Confocal microscopic image observation in Figure 2b revealed that the untreated control group showed high red J-aggregate fluorescence, suggesting that mitochondria were still intact and that there was a high level of Ψm compared to the treated cells. Meanwhile, the treated sample showed a decrease in a concentration-dependent manner in the red J-aggregated fluorescence, supporting the decrease observed in Figure 2a. The reduction in J-aggregated fluorescence reflects a concentration-associated trend rather than a uniformly strong concentration-dependent effect across all concentrations. At low concentration (8 μg/mL), we observed a modest decrease (4% reduction) and a more pronounced decrease (15% reduction) at the highest concentration (32 μg/mL).

### 2.3. VCF7 Induces Chromatin Condensation and Nuclear Fragmentation (DAPI Staining)

**Figure 3:**
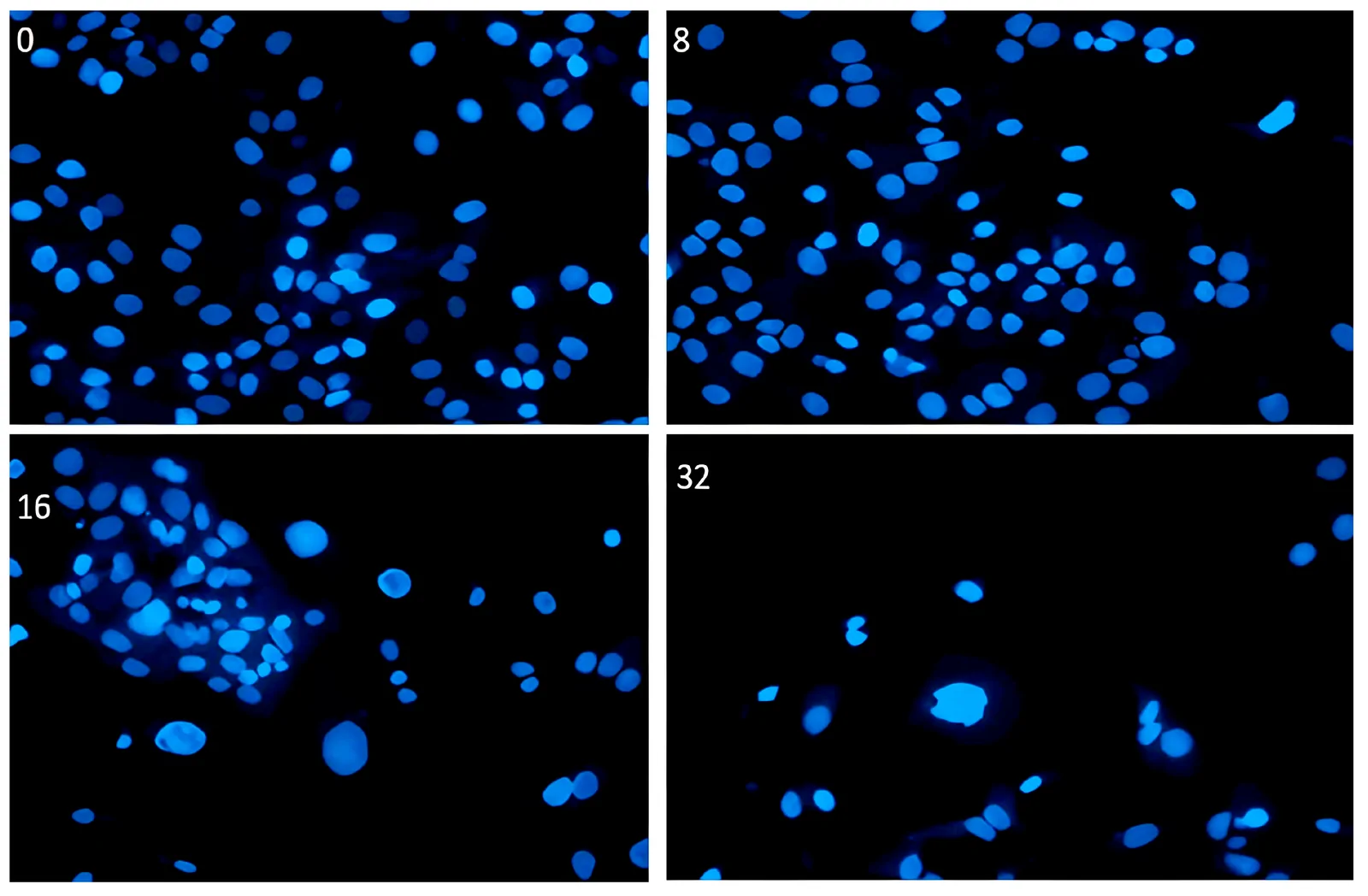
Representative fluorescence images of DAPI-stained OVCAR-3 cells treated with VCF7 at 0, 8, 16, and 32 µg/mL, respectively. Fluorescence images were captured under 20× optical resolution of the microscope.

DAPI staining was used to analyze chromatin condensation and nuclear structure alterations in OVCAR-3 cells treated with VCF7 for 48 h (Figure 3). The nuclei of control cells appeared homogeneously stained, with even and well-defined morphology and a uniformly distributed blue fluorescence, indicating their normal structural organization. Treatment with VCF7 resulted in dose-concentration-dependent and gradual nuclear structure alterations. At a concentration of 8 µg/mL, initial signs of chromatin condensation became evident in some cells. At concentrations of 16 and 32 µg/mL, intense hyperfluorescence, chromatin condensation, and nucleus fragmentation occurred in most of the cells with the formation of apoptotic bodies, i.e., small membrane-bounded fragments of the cell nucleus, which mark the final step of apoptosis.

### 2.4. VCF7 Induces Internucleosomal DNA Fragmentation

**Figure 4:**
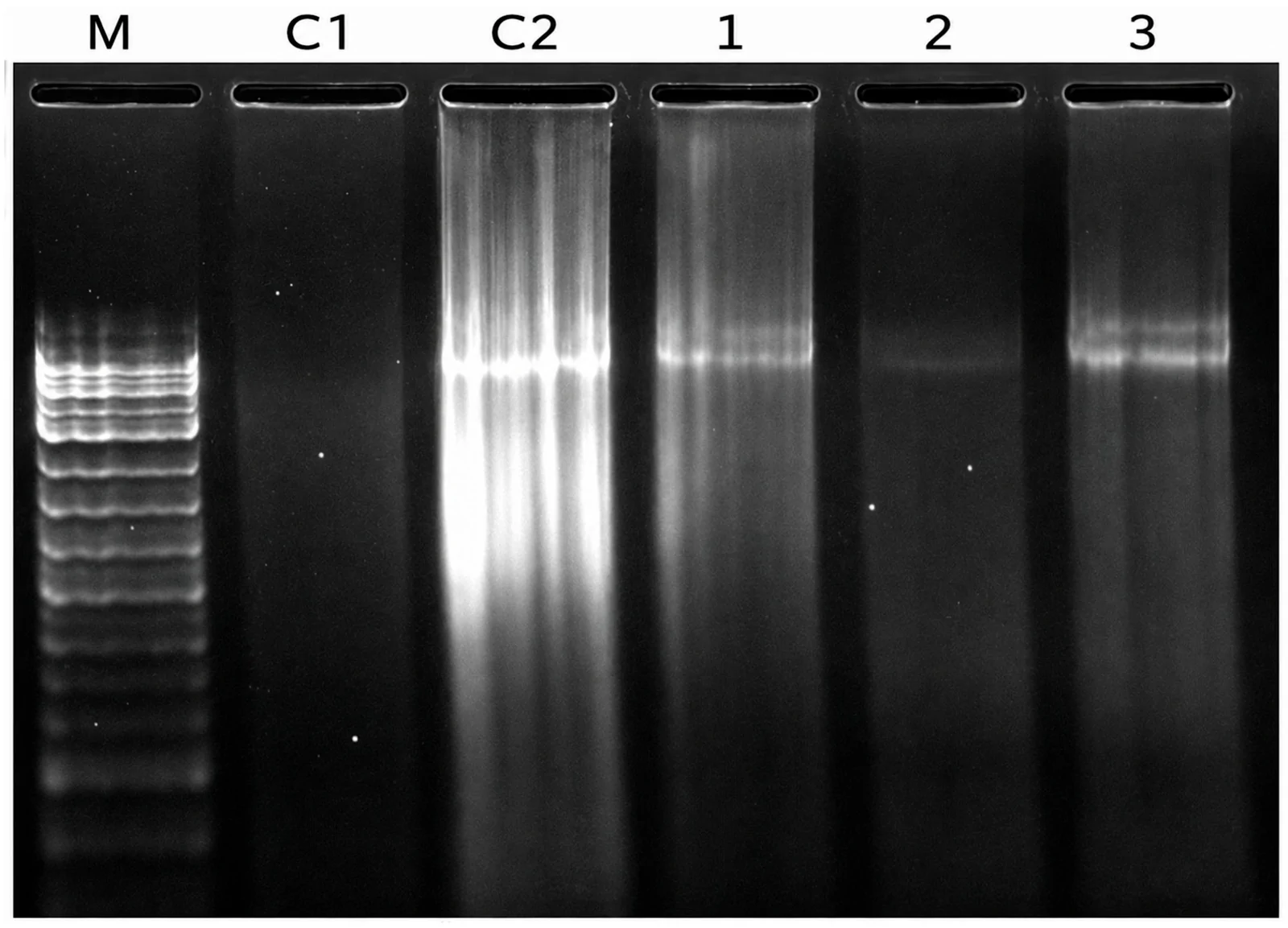
DNA fragmentation image by agarose gel electrophoresis stained with ethidium bromide. OVCAR-3 cells were treated with VCF7 at 0, 8, 16, and 32 µg/mL. Lane (M) DNA molecular weight marker, Lane (C1) 0 µg/mL of OVCAR-3, Lane (C2) positive control or OVCAR-3 cells treated with camptothecin to induce apoptosis. Lanes 1, 2, and 3 represent the OVCAR-3 cells treated with 8, 16, and 32 µg/mL of VCF7, respectively.

To assess whether VCF7 induces apoptotic DNA degradation, total genomic DNA was isolated from OVCAR-3 cells treated with VCF7 (0, 8, 16, and 32 μg/mL) and camptothecin (as a positive control) for 48 h. The untreated OVCAR-3 cells possessed one major high-molecular-weight DNA band without any fragmentation, demonstrating intact genome integrity (Figure 4). Camptothecin-treated cells used as a positive control showed a characteristic ladder pattern associated with effective internucleosomal DNA fragmentation (Figure 4). The treated OVCAR-3 cells showed concentration-dependent DNA fragmentation. At 8 µg/mL, VCF7 showed weak DNA fragmentation characterized by the appearance of a faint DNA smear. At 16 µg/mL, VCF7 treatment resulted in DNA degradation characterized by the formation of a smear pattern with discrete low-molecular-weight bands. At 32 µg/mL VCF7, a high degree of DNA fragmentation was shown, characterized by a clear smear with only remnants of high-molecular-weight genomic DNA (Figure 4). Notably, a combination of discrete ladders and smeared DNA fragments observed in cells treated with VCF7 at higher concentrations can be considered as evidence for concurrent apoptosis and secondary necrosis. This finding aligns well with Annexin V/PI results showing the simultaneous existence of apoptotic and necrotic populations at 16–32 µg/mL VCF7.

### 2.5. VCF7 Activates Caspase-3 in OVCAR-3 Cells

**Figure 5:**
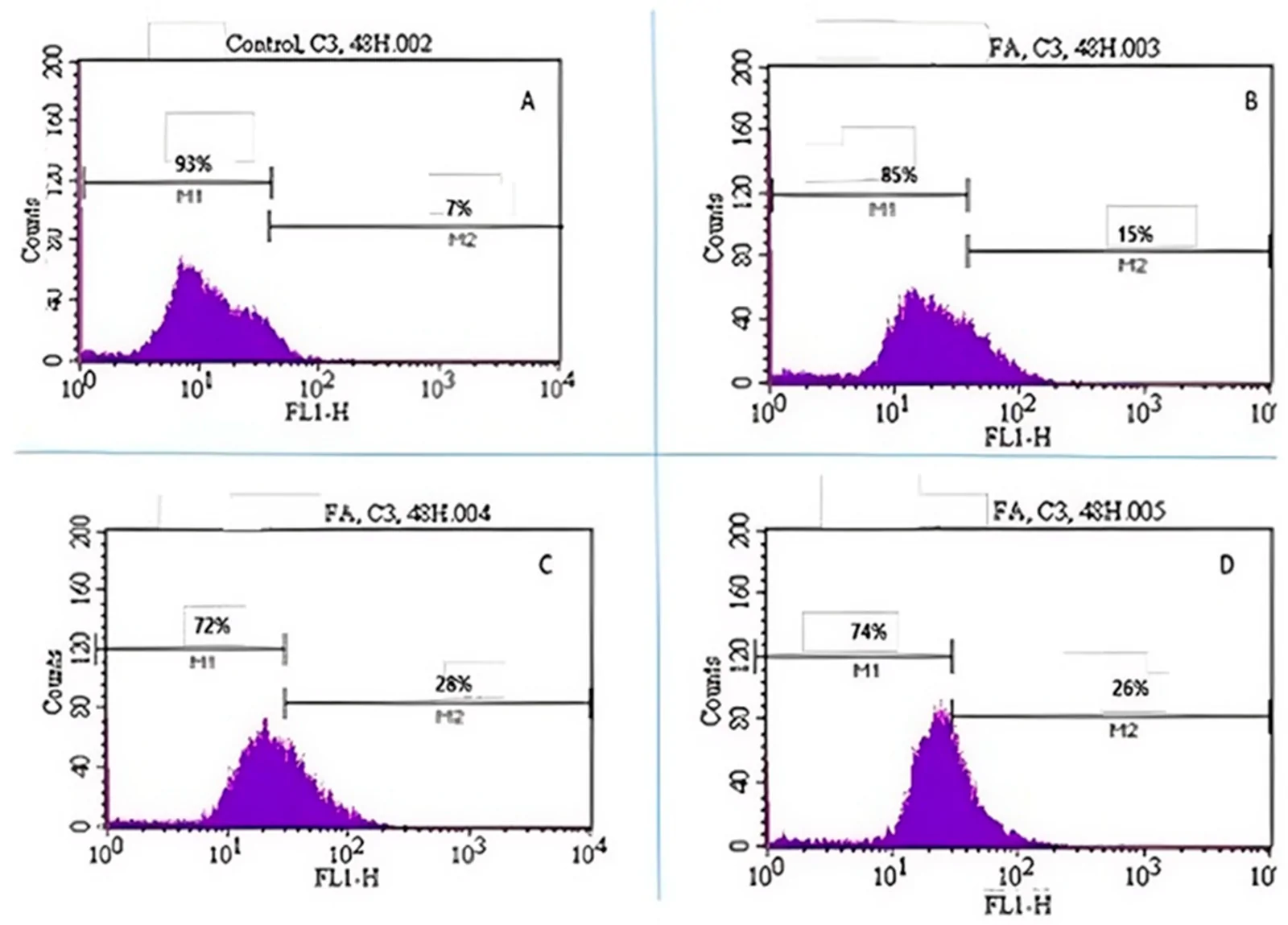
Representative flow cytometric analysis of caspase-3 assay in OVCAR-3 cells following 48 h treatment. (**A**) control; (**B**) 8 µg/mL; (**C**) 16 µg/mL; (**D**) 32 µg/mL. Histogram showed (M1) representing the distribution negative caspase-3 of the OVCAR-3 cells. Meanwhile, (M2) represent the distribution positive caspase-3 cells.

**Figure 6:**
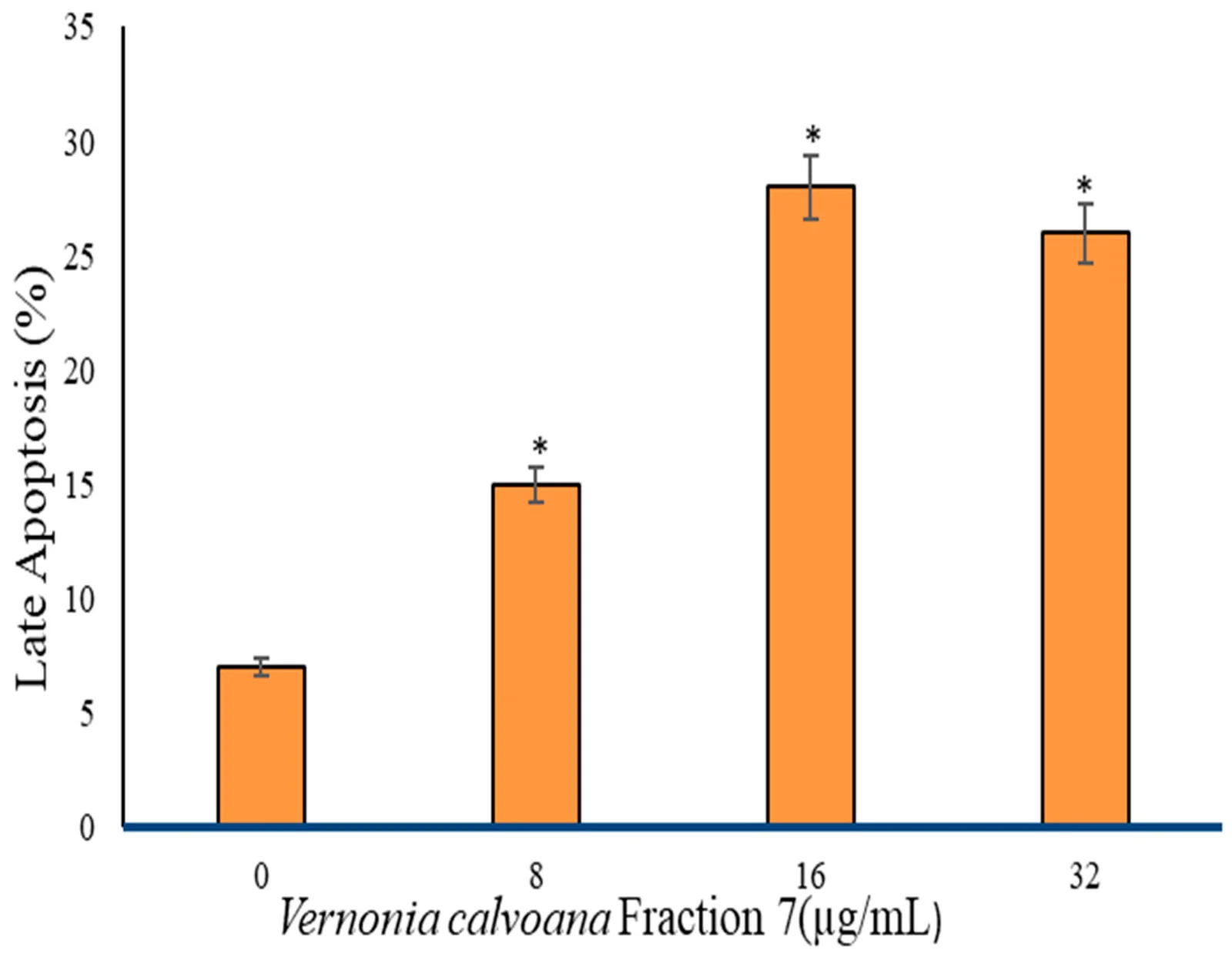
Percentages of apoptotic cells after treatment of OVCAR-3 cells with VCF7. Each data point represents the mean value and standard deviation of three independent experiments. * Denotes a statistically significant difference (*p* < 0.05) between the control and the treated groups.

Flow cytometric quantification of caspase-3-positive cells was performed following 48 h of VCF7 treatment at 0, 8, 16, and 32 μg/mL. Figure 5 and Figure 6 show that the percentage of late-apoptotic caspase-3-positive (M2) cells increased with VCF7 treatment in a dose-concentration-dependent manner: 7% (control), 15% (8 μg/mL), 28% (16 μg/mL), and 26% (32 μg/mL). A strong dose-concentration–response relationship in caspase-3 activation was observed (*p* < 0.05). The peak caspase-3 activation was at 16 μg/mL, followed by a marginal reduction at 32 μg/mL, which is consistent with the IC_50_ threshold (~18.56 μg/mL) previously obtained [25]. Western blot analysis confirmed a concentration-dependent upregulation of caspase-9 protein levels, providing additional evidence for engagement of the intrinsic mitochondrial apoptotic cascade.

### 2.6. VCF7 Modulates Apoptotic Regulatory Proteins and mRNAs (Western Blot and qRT-PCR)

#### 2.6.1. Modulation of Apoptosis-Related Genes in OVAR-3 Cells Treated with VCF7

**Figure 7:**
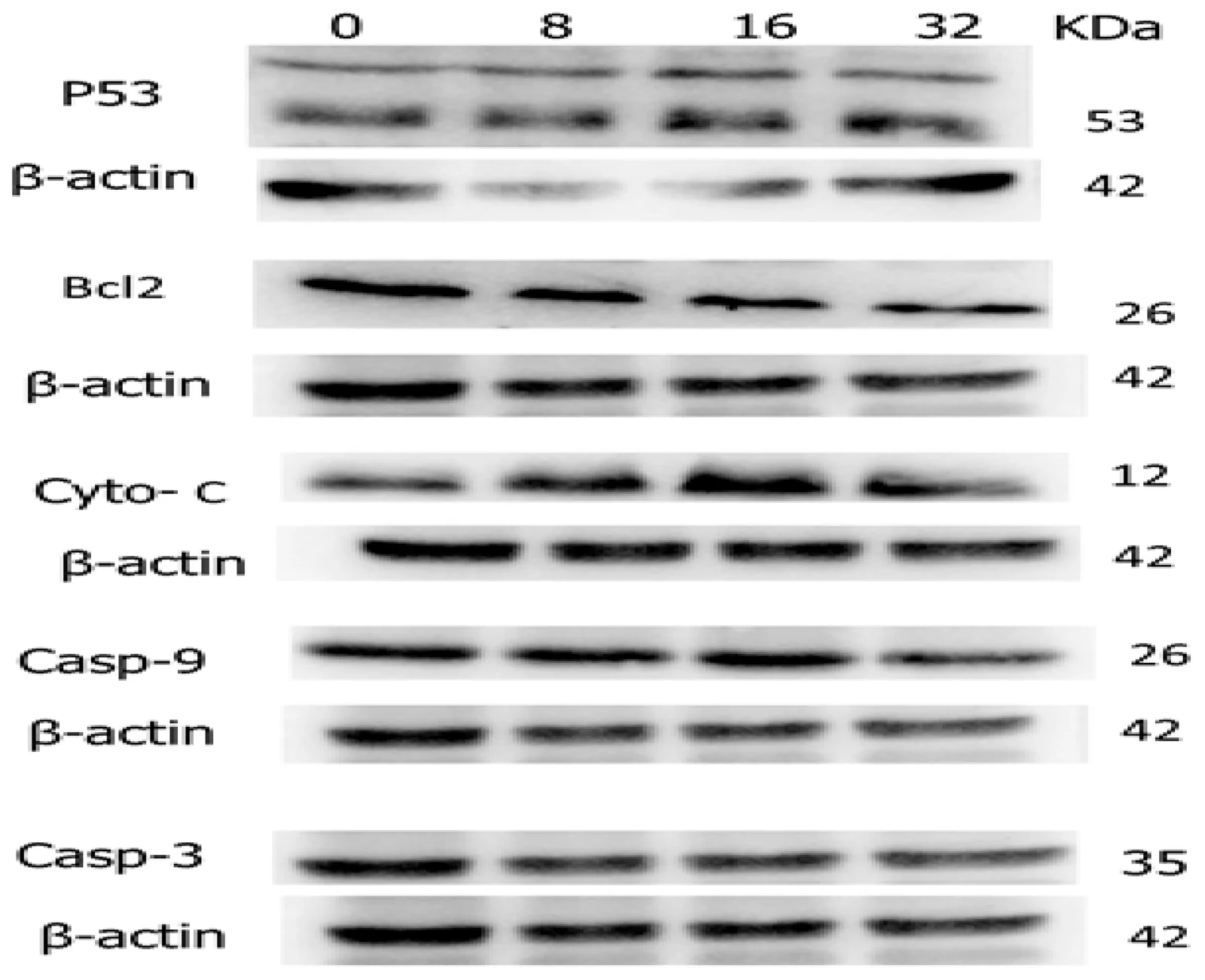
Western blot images of P53, Bcl-2, cytochrome c, caspase-9, and caspase-3 protein expression in treated and untreated cells. β-actin served as a loading control.

**Figure 8:**
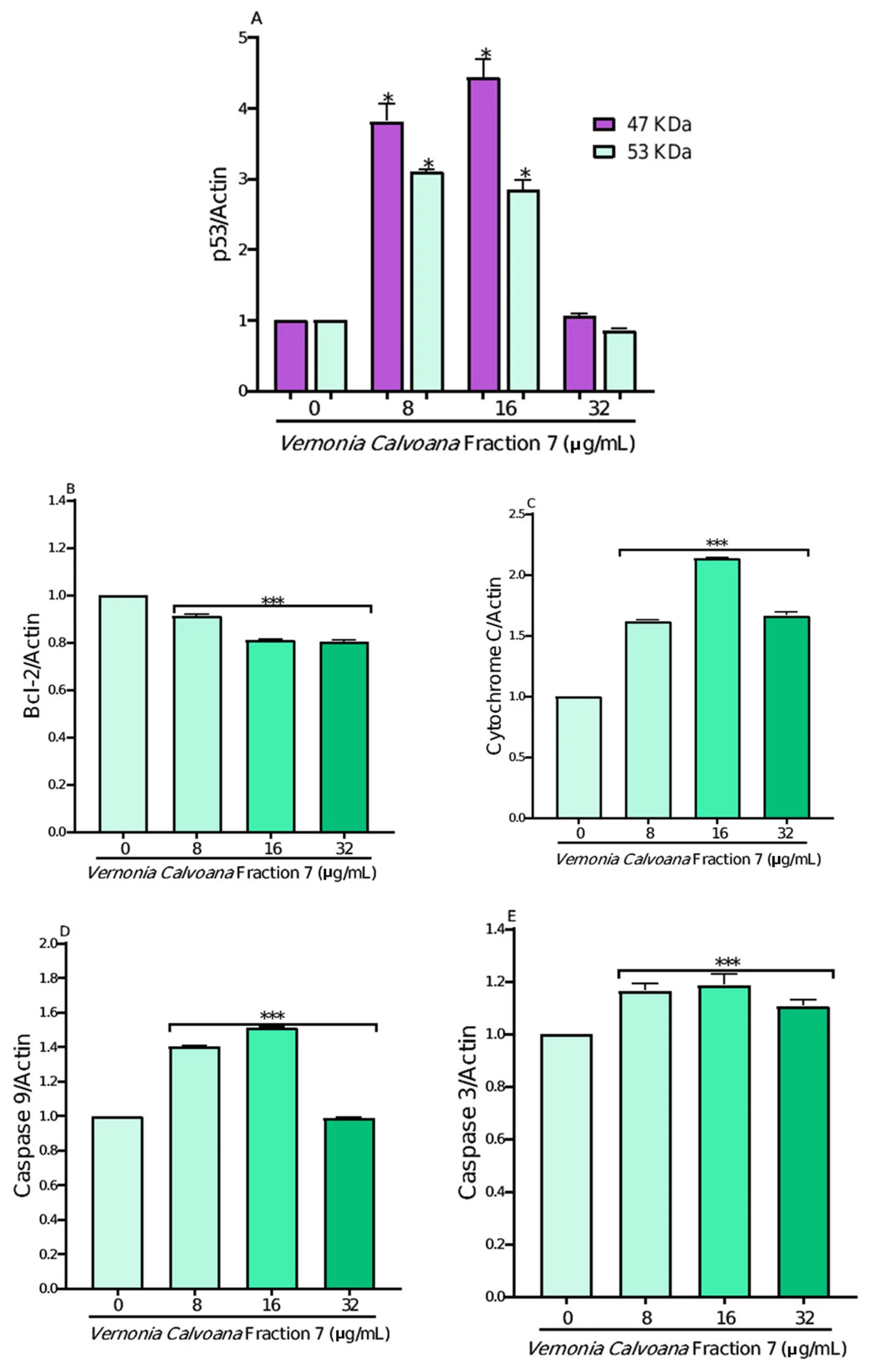
VCF7 induces apoptosis-related protein modulation in OVCAR-3 cells. (**A**–**E**) Quantitative analysis of Western blot band intensities showing protein expression levels of (**A**) p53, (**B**) Bcl-2, (**C**) cytochrome c, (**D**) caspase-9, and (**E**) caspase-3 in OVCAR-3 cells following treatment with varying concentrations of *Vernonia calvoana* fraction seven (VCF7). Protein expression was normalized to β-actin (loading control) and expressed relative to the control group (0 µg/mL VCF7). Data are presented as mean ± SD (*n* = 3). Statistical analysis was performed using one-way ANOVA followed by Dunnett’s post hoc test. * *p* < 0.05; *** *p* < 0.001 vs. control.

Western blot was performed to investigate the expression levels of essential proteins involved in apoptotic regulation in OVCAR-3 cells after treatment with *Vernonia calvoana* fraction seven (VCF7), as shown in Figure 7. The Western blot assay was carried out using antibodies against p53 (47/53 kDa), Bcl-2 (26 kDa), cytochrome c (12 kDa), caspase-9 (45 kDa), and caspase-3 (35 kDa) in addition to the housekeeping control protein β-actin (42 kDa). Concentration-dependent variations were observed in the expression profiles of all apoptotic regulatory proteins after treatment with VCF7. The tumor suppressor p53 showed increasing expression levels (*p* < 0.05) as the VCF7 concentrations (Figure 8A). This is because of its established role as the transcriptional factor activating pro-apoptotic genes in response to genotoxic stresses. On the other hand, Bcl-2, which acts as an anti-apoptotic protein maintaining mitochondrial membrane integrity, was consistently downregulated as the VCF7 concentrations increased (*p* < 0.001), leading to a shift in the balance between Bcl-2 and pro-apoptotic proteins toward pro-apoptosis (Figure 8B). Also, increasing concentrations of VCF7 led to a significant increase in cytochrome c protein levels (*p* < 0.001) in OVCAR-3 cells, consistent with cytochrome c translocation from the mitochondrial intermembrane space as part of mitochondrial outer membrane permeabilization induced by the extract (Figure 8C). This analysis was performed on total protein lysates (RIPA extraction) without cytosolic/mitochondrial subcellular fractionation. Therefore, the findings reflect modulation of total cytochrome c levels rather than confirmed cytosolic release. The expression levels of caspase-9 were gradually increased (*p* < 0.001) based on VCF7 concentrations, supporting the activation of the intrinsic apoptotic machinery prior to the terminal execution stage (Figure 8D). Importantly, a significant concentration-dependent increase in caspase-3 expression (*p* < 0.001) was evident in OVCAR-3 cells, particularly at 16 and 32 µg/mL, compared to the control group (Figure 8E). Upregulation in the expression of caspase-3, which is the primary executioner caspase breaking down cellular structures, represents the convergence of the mitochondrial pathway, which results in activation of caspases via the cytochrome c-induced activation of apoptosomes and caspase-9. Collectively, VCF7 downregulation modulates the expression levels of p53, Bcl-2, cytochrome c, caspase-9, and caspase-3 proteins, providing convincing evidence of its action on the intrinsic mitochondrial pathway in the apoptosis of OVCAR-3 ovarian cancer cells (Figure 8).

#### 2.6.2. qRT-PCR Gene Expression Analysis

**Figure 9:**
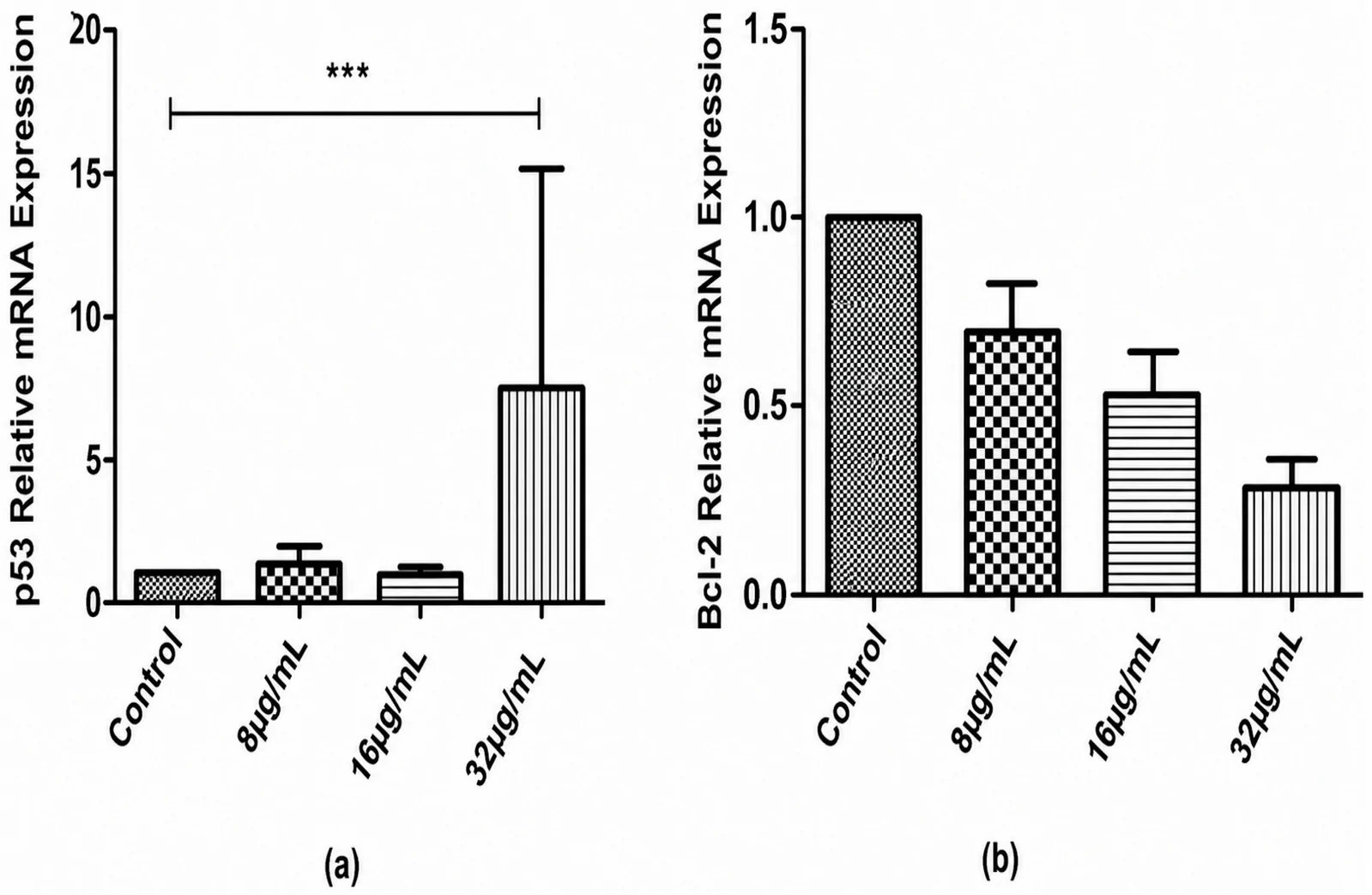
Expression levels of p53 and Bcl-2 mRNAs in OVCAR-3 cells. Relative mRNA expression levels of p53 in (**a**) and relative mRNA expression levels of Bcl-2 in (**b**). p53 and Bcl-2 were measured by RT-qPCR after exposure of OVCAR-3 cells to VCF7 at different concentrations of 0 (control), 8, 16, and 32 μg/mL. p53 mRNA expression was significantly elevated only at 32 μg/mL, whereas Bcl-2 mRNA expression was gradually decreased at increasing concentrations of VCF7. Statistical significance is denoted as, *** *p* < 0.001.

Quantitative real-time PCR was performed to assess whether VCF7 modulates the transcription of key apoptotic regulatory genes, as shown in Figure 9a,b panels. The mRNA expression of p53 was shown to be significantly increased in a concentration-dependent manner, with the highest expression observed at 32 μg/mL (*p* < 0.001). In addition, the mRNA levels of Bcl-2 were gradually decreased, implying transcriptional repression. In summary, based on the results obtained using qRT-PCR, it can be concluded that VCF7 mediates the activation of a transcriptionally induced apoptosis pathway involving the p53 protein, which is associated with a decrease in the expression level of Bcl-2 transcripts.

## 3. Discussion

The present study provides mechanistic evidence that VCF7 induces apoptosis in OVCAR-3 human ovarian cancer cells through the intrinsic mitochondrial pathway. Using complementary in vitro assays, we demonstrate a coherent and inter-reinforcing mechanistic sequence as follows: VCF7-induced mitochondrial membrane depolarization → cytochrome c expression → p53/Bcl-2 transcriptional reprogramming → caspase-9 cascade activation → internucleosomal DNA fragmentation, chromatin condensation, nuclear fragmentation, and apoptotic body formation. These findings extend and mechanistically anchor our previous publication, demonstrating that VCF7 inhibits OVCAR-3 cell proliferation via oxidative stress, DNA damage, and S-phase cell cycle arrest [25].

Loss of mitochondrial membrane potential is a critical early indicator of intrinsic apoptotic commitment and serves as the point of no return in the commitment of cells to caspase-dependent cell death [29,35]. JC-1 data demonstrated a statistically significant, concentration-dose-dependent decrease of Ψm in VCF7-treated OVCAR-3 cells, confirmed by both fluorometry and confocal microscopy. This finding is consistent with the earlier observation that VCF7 induces oxidative stress in these cells, evidenced by elevated malondialdehyde (MDA) production and depletion of the antioxidant enzymes catalase and glutathione peroxidase [25]. Reactive oxygen species (ROS) accumulation disrupts the electron transport chain at the inner mitochondrial membrane, oxidizes cardiolipin (a mitochondria-specific phospholipid required for cytochrome c binding), and directly promotes apoptosis [36]. Analogously, cisplatin-induced mitochondrial dysfunction in leukemia cells was attributed to Ψm depolarization driven by ROS-mediated disruption of the electron transport chain [37], and arsenic trioxide was shown to engage mitochondrial apoptosis in HL-60 cells through oxidative stress and caspase-3/Ψm-dependent pathways [37]. The data revealed that VCF7-induced oxidative stress is the upstream driver that destabilizes the mitochondrial membrane and initiates the intrinsic apoptotic cascade in OC cells.

The central role of the Bcl-2 protein family in regulating MOMP and governing the apoptotic fate decision is well established [38]. Bcl-2 prevents apoptosis by preserving mitochondrial membrane integrity and blocking Bax/Bak oligomerization; when overwhelmed, Bax translocates to the outer mitochondrial membrane, oligomerizes into pores, and enables cytochrome c release [38,39]. Our Western blot data demonstrate that VCF7 downregulates Bcl-2 expression in a concentration-dependent manner at the protein level, consistent with relief of the mitochondrial apoptotic block. qRT-PCR further confirmed Bcl-2 transcriptional repression, indicating that VCF7 acts at the gene expression level to suppress this anti-apoptotic guardian. These findings parallel those reported for other plant-derived anticancer agents: curcumin (from *Curcuma longa*) downregulated Bcl-2 and upregulated Bax in ovarian cancer cells [40,41]; *Vernonia amygdalina* extract induced Bcl-2 suppression and Bax upregulation in breast cancer cells [42]; and luteolin (from various medicinal plants) activated the mitochondrial apoptotic pathway in OVCAR-3 cells through a similar Bcl-2/Bax mechanism [43].

The tumor suppressor p53, the most frequently mutated gene in human cancer, functions as a transcriptional activator of pro-apoptotic targets (Bax, PUMA, and Noxa) and a repressor of Bcl-2 in response to DNA damage and cellular stress [32]. The concentration-dependent p53 upregulation observed at both the protein and transcript levels in VCF7-treated OVCAR-3 cells is consistent with p53 activation driven by VCF7-induced DNA damage, as we previously demonstrated by comet assay [25]. Together, p53 protein upregulation and Bcl-2 mRNA downregulation establish that VCF7’s apoptotic activity originates, at least in part, from a p53-dependent transcriptional response to DNA damage and oxidative stress, which amplifies the mitochondrial apoptotic cascade through Bcl-2 suppression.

Caspase-3 activation is required for the execution phase of apoptosis, including cytoskeletal dismantling. Our flow data demonstrate statistically significant caspase-3 activation in VCF7-treated cells, peaking at 16 μg/mL (28% caspase-3 positive cells vs. 7% in controls). Western blot analysis demonstrates that VCF7 also leads to an increase in caspase-9, indicating caspase-9 involvement in upstream processes leading to caspase activation in the apoptosome. The direct involvement of caspase-9 activation serves to tie the mitochondrial events (increased cytochrome c levels, caspase-9 recruitment to form the apoptosome) to the subsequent action of the executioner cascade (chromatin condensation/internucleosomal DNA fragmentation).

DNA fragmentation via agarose gel electrophoresis serves as the last biochemical step that signifies caspase-induced apoptosis, which involves the enzymatic cleavage of chromosomal DNA in linker areas using CAD to generate fragments of ~180–200 bp and multiples thereof [27,28]. The partial ladder formation with DNA smearing in our study, as demonstrated in our gel images (Figure 4), is indicative of a simultaneous activation of apoptosis and secondary necrosis due to excessive cell death induced by higher doses of VCF7. Indeed, partial DNA laddering is commonly reported in cases when DNA fragmentation is accompanied by necrosis and when cells are treated above the IC_50_ concentrations, leading to rapid cell death when non-specific, random breaks occur [44].

In conclusion, given the dramatic increase in ovarian cancer incidence, especially in Africa and Asia, as well as ongoing racial disparities in OC-related outcomes in the United States, new effective therapy options that are more accessible and cheaper are needed. Since *Vernonia calvoana* has been safely consumed as a leafy vegetable in West and Central Africa for centuries, there is reason to believe that its active constituents will be well tolerated. Further experiments should focus on the following areas: (i) isolation and structure identification of the active phytochemicals; (ii) pharmacokinetics and toxicity testing in vitro and in vivo; (iii) investigation of possible synergies with the standard of care chemotherapy (carboplatin, paclitaxel); and (iv) efficacy evaluation of VCF7 in OC xenograft mouse models, especially in the context of obesity, which adversely impacts OC outcomes.

## 4. Materials and Methods

### 4.1. Vernonia calvoana Preparation and Fractionation

Fresh leaves of *Vernonia calvoana* were harvested in Bangou, Cameroon, rinsed, and air-dried in sunlight for 24 h, followed by shade-drying for 5 days. Five hundred grams of dried powdered leaves were macerated in 600 mL of methanol and heated at 50 °C. The mixture was filtered through Whatman No. 1 filter paper and concentrated to dryness using a rotary evaporator (Marshall Scientific, Hampton, NH, USA). The crude extract was further air-dried under a fume hood until the methanol residual was completely removed and was stored at 4 °C until fractionation. Column chromatography fractionation was performed according to previously described bioassay-guided methods [42,43], yielding 10 fractions. Fraction seven (VCF7), selected for its highest antiproliferative activity toward OVCAR-3 cells (IC_50_ = 18.56 μg/mL) [24], the dry VCF7 was dissolved in DMSO to prepare the stock solution, which was then diluted in cell culture media to reach the final treatment concentration, keeping the DMSO concentration at ≤0.1% *v*/*v* across all conditions and well below levels known to affect cell viability.

### 4.2. Cell Culture and Treatment

Human ovarian adenocarcinoma OVCAR-3 cells (ATCC, Manassas, VA, USA) were cultured in RPMI-1640 medium supplemented with 20% heat-inactivated fetal bovine serum (FBS) and 1% penicillin/streptomycin (Thermo Scientific, Waltham, MA, USA) at 37 °C in a humidified 5% CO_2_ atmosphere. The medium was changed every 2–3 days. At 80% confluency, cells were trypsinized with 0.25% trypsin–0.03% EDTA, counted, and seeded into appropriate plate formats. Cells were treated with VCF7 at concentrations of 0, 8, 16, and 32 μg/mL for 48 h.

### 4.3. Annexin V/Propidium Iodide Apoptosis Assay

OVCAR-3 cells (5 × 10^5^ cells/well in 6-well plates) were treated with VCF7 for 48 h, harvested by trypsinization, washed twice with cold PBS, and resuspended in 1× Annexin V binding buffer at 10^6^ cells/mL. Cells were incubated with 5 μL Annexin V-FITC and 5 μL PI (BD Biosciences, Annexin V Apoptosis Detection Kit) for 15 min at room temperature in the dark. Dot-plot analysis was performed on a Cellometer Vision flow cytometer using FCS Express 4 software (De Novo Software, Nexcelom, BSI, TX, USA). Cell populations were classified as viable or live (Annexin V^−^/PI^−^), early apoptotic (Annexin V^+^/PI^−^), late apoptotic (Annexin V^+^/PI^+^), and necrotic (Annexin V^−^/PI^+^). Experiments were performed in triplicate.

### 4.4. Mitochondrial Membrane Potential Assay (JC-1)

Mitochondrial membrane potential was assessed using the JC-1 Assay Kit (Cayman Chemical, Ann Arbor, MI, USA). OVCAR-3 cells (5 × 10^5^ cells/well) were treated with VCF7 for 48 h. Cells were washed with PBS, incubated with JC-1 dye (2 μM) at 37 °C for 20 min, and washed twice with assay buffer. Fluorescence was measured using a BioTek microplate reader (Agilent BioTek, Santa Clara, CA, USA) (J-aggregates: excitation/emission 535/590 nm; JC-1 monomers: 475/530 nm). Results were expressed relative to the control. Confocal fluorescence microscopy images (20×) were acquired to confirm the fluorometric data. Statistical significance was evaluated by one-way ANOVA with Dunnett’s post hoc test (*p* < 0.05). Experiments were performed in triplicate.

### 4.5. DAPI Staining for Nuclear Morphology

OVCAR-3 cells seeded on glass coverslips were treated with VCF7 for 48 h, washed with PBS, fixed in 4% paraformaldehyde (15 min, room temperature), and permeabilized with 0.1% Triton X-100 in PBS (5 min). Cells were stained with DAPI solution (0.5 μg/mL, 10 min, in the dark, at room temperature), washed, and mounted on glass slides. Fluorescence images were captured at 20× magnification using an Olympus fluorescence microscope (Cambridge Technology, Lexington, MA, USA). Nuclei displaying condensation, fragmentation, or apoptotic body formation were scored as apoptotic. All images were representative of ≥3 independent experiments.

### 4.6. DNA Ladder Assay (Internucleosomal DNA Fragmentation)

To detect internucleosomal DNA fragmentation, total genomic DNA was extracted from VCF7-treated and untreated OVCAR-3 cells using the Apoptotic DNA Ladder Kit (Sigma-Aldrich, Cat. #1183524600, St. Louis, MO, USA) according to the manufacturer’s protocol. Camptothecin (5 μM, 24 h)-treated OVCAR-3 cells served as a positive control for apoptotic DNA laddering. Briefly, cells (5 × 10^6^ cells/well in 6-well plates) were treated with VCF7 at 0, 8, 16, and 32 μg/mL for 48 h, harvested by trypsinization, and lysed. DNA was precipitated, washed, and resuspended in TE buffer. DNA concentration was determined by NanoDrop spectrophotometry. Equal amounts of DNA (5 μg per lane) were resolved on a 1.5% agarose gel containing ethidium bromide (0.5 μg/mL) by electrophoresis at 80 V for 90 min. Gels were visualized under UV illumination and photographed. A 100-bp DNA molecular weight ladder was included in all gels to facilitate fragment size determination. Experiments were performed in triplicate.

### 4.7. Caspase-3 Activity Assay

Caspase-3 activity was quantified by flow cytometry using BD Pharmigen^TM^ FITC Active Caspase-3 Apoptosis kit (Cat# 571606, BD Biosciences, San Jose, CA, USA). OVCAR-3 cells (5 × 10^5^ cells/well) were treated with VCF7 for 48 h, harvested, and fixed in 70% ice-cold methanol (30 min, 4 °C). Fixed cells were permeabilized and stained with an anti-active caspase-3-FITC antibody per the manufacturer’s protocol. Cells were analyzed by flow cytometry. The percentage of caspase-3-positive cells (M2 region) was recorded. Three independent experiments were performed.

### 4.8. Western Blot Analysis

Total protein was extracted using RIPA buffer with a protease/phosphatase inhibitor cocktail (Sigma-Aldrich). Protein concentration was determined by Bradford assay. Thirty micrograms of protein per lane were resolved on 10–12% SDS-PAGE gels and transferred to nitrocellulose membranes. Membranes were then incubated in a blocking buffer of 5% nonfat milk in TBST for 1 h at room temperature, followed by incubation with primary antibodies for p53 (47/53 kDa), Bcl-2 (26 kDa), cytochrome c (12 kDa), cleaved caspase-9 (45 kDa), and β-actin (42 kDa) overnight at 4 °C (Cell Signaling Technology, Danvers, MA, USA). Detection was accomplished using HRP-conjugated secondary antibodies and ECL. Band density measurements were determined.

### 4.9. Quantitative Real-Time PCR (qRT-PCR)

Total RNA was isolated from VCF7-treated cells using TRIzol (Thermo Fisher Scientific, Waltham, MA, USA) and quantified by NanoDrop (A260/A280 ≥ 1.8). Two micrograms of RNA were reverse-transcribed using the High-Capacity cDNA Reverse Transcription Kit (Cat# 4374967, Applied Biosystems, Waltham, MA, USA). qRT-PCR was performed using SYBR Green Master Mix (Applied Biosystems) with gene-specific primers for p53, Bcl-2, and β-actin on a Step OnePlus system. The primers used for amplification of p53 and Bcl-2 transcripts are as follows: p53 forward GCCACAGGACAGGCTGAC, reverse GCTGCTGCTGCTGCTGTT; Bcl-2 forward GGTGGGATGACCTGGTG and reverse TCTCCTCCTTCTTCCGTTG. Thermal cycling conditions consisted of an initial denaturation at 95 °C for 10 min, followed by 40 cycles of 95 °C for 15 s and 60 °C for 60 s with melt-curve analysis (60–95 °C) performed afterward to confirm primer specificity. Primer efficiency was validated using a standard curve from serial cDNA dilution prior to use in relative quantification (efficiency 90–110%, R^2^ > 0.98). Relative mRNA expression was calculated using the 2^−^ΔΔCt method, normalized to β-actin. All reactions were performed in triplicate.

### 4.10. Statistical Analysis and Software

Data were expressed as mean ± standard deviation (SD) from at least three independent experiments (*n* = 3). Statistical comparisons were performed using one-way ANOVA with Dunnett’s post hoc test (GraphPad Prism 9, San Diego, CA, USA). *p* < 0.05 or 0.001 was considered statistically significant. Image J (v0.6.0) was used to analyze the band intensity of the Western blot. BioRender was used to design the summary of the apoptotic pathways.

## 5. Conclusions

This research presents mechanistic evidence indicating that VCF7, the most bioactive component of the edible West African medicinal plant *Vernonia calvoana*, triggers apoptosis in human OVCAR-3 ovarian cancer cells through the intrinsic mitochondrial pathway. The multi-assay mechanistic profile established in this study included mitochondrial membrane potential (ΔΨm) depolarization, cytochrome c translocation, p53 activation, Bcl-2 downregulation, caspase-9 activation, internucleosomal DNA fragmentation, chromatin condensation, and the formation of apoptotic bodies, which marks a significant progression from our earlier report that demonstrated VCF7’s antiproliferative, oxidative stress, DNA-damaging, and cell cycle-arresting effects in the same cellular model.

**Figure 10:**
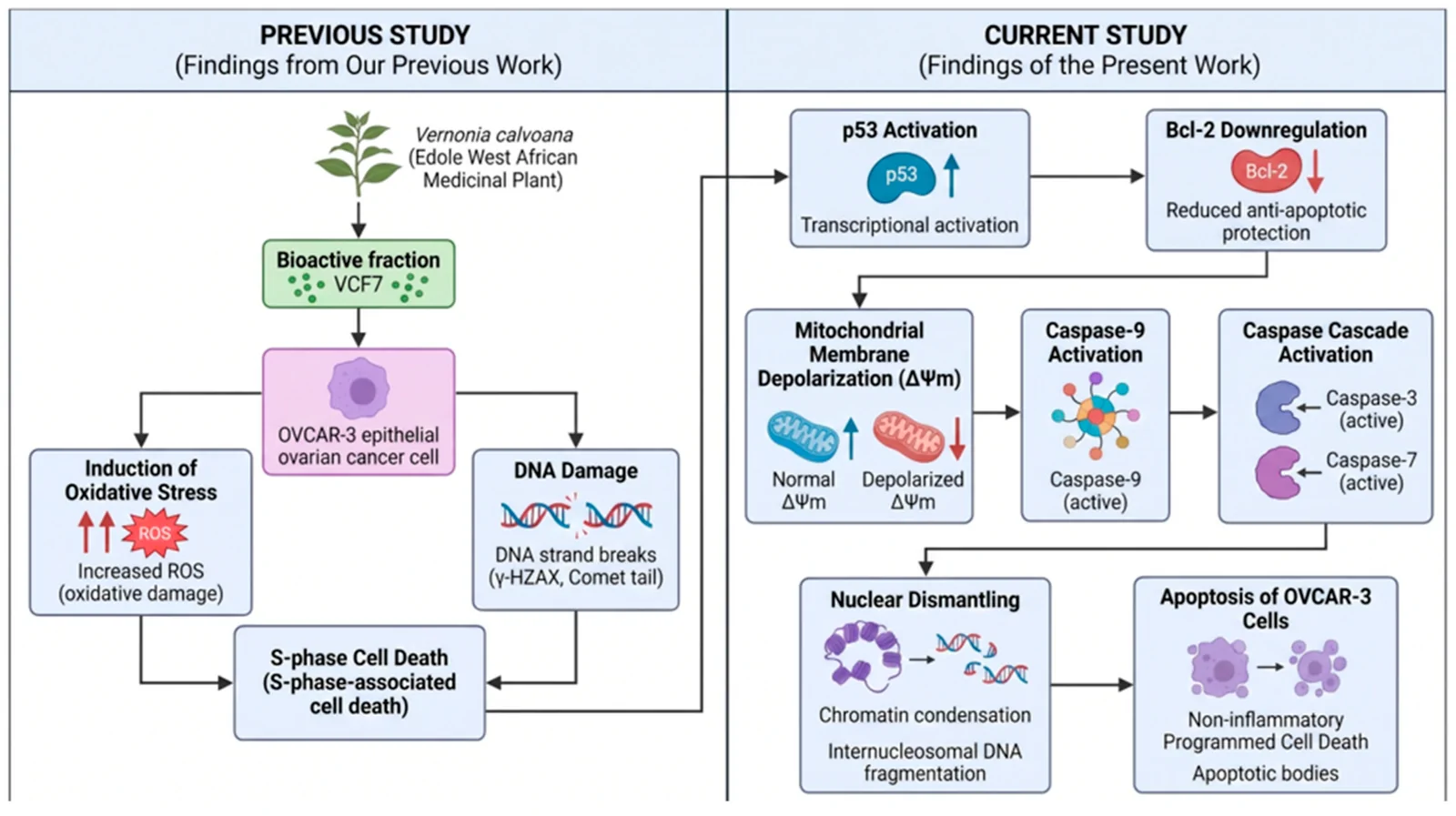
Integrated mechanistic sequence of VCF7-induced apoptosis in OVCAR-3. The (**left panel**) summarizes findings from our previous study Mbemi et al. 2020, showing that *Vernonia calvoana* induced oxidative stress, DNA damage, and cell cycle arrest in S phase [25]. The (**right panel**) presents findings from the current study, demonstrating that *Vernonia calvoana* activates p53, downregulates Bcl-2, induces mitochondrial membrane depolarization (ΔΨm), and activates caspase-9 and the caspase-3/7 cascade, leading to chromatin condensation, internucleosomal DNA fragmentation, and apoptotic cell death in OVCAR-3 cells.

Collectively, based on our previously published data (Mbemi et al. (2020) [25]), these results outline a mechanistic sequence of VCF7-induced oxidative stress → DNA damage → p53 activation → Bcl-2 suppression → ΔΨm depolarization → cytochrome c translocation → caspase-9 activation → nuclear disassembly and apoptotic cell death as shown in Figure 10 using BioRender.

Considering the anticipated global rise in ovarian cancer cases. It is projected to reach approximately 500,000 new cases annually by 2050, and the ongoing racial disparities in clinical outcomes, along with plant-derived compounds like VCF7, which exhibit multi-targeted and potentially low-toxicity anticancer properties, represent promising and urgently needed additions to the therapeutic arsenal. Future research should prioritize the identification of active phytochemical constituents, pharmacokinetic and toxicological profiling, and the validation of efficacy in relevant in vivo models, including murine systems.

## Data Availability

The data that support the present research study are included in the article.

## Abbreviations

The following abbreviations are used in this manuscript:

Ψm	Mitochondrial Membrane Potential Assay
V/PI	Annexin V/Propidium Iodide
qRT-PCR	Quantitative Real-Time PCR
